# Supplementary material for: Patterns of Intron Gain and Loss in Fungi
Source: PLoS Biol. 2004 Nov 30;2(12):e422. doi: 10.1371/journal.pbio.0020422 (PMC532390; doi:10.1371/journal.pbio.0020422)
Supplement: Table S1 — Also available at http://genes.mit.edu/NielsenEtAl/. (4.3 MB ZIP). [file pbio.0020422.st001.zip › NielsenEtAl/html/34.html]

AN4576.1.NCU08315.1.MG04166.1.FG05063.1


```
 CLUSTAL W (1.82) Multiple Sequence Alignments - Introns Inserted


Sequence 1: MG04166.1	446 aa
Sequence 2: FG05063.1	440 aa
Sequence 3: NCU08315.1	445 aa
Sequence 4: AN4576.1	459 aa
Alignment Length: 466 aa
Number Identitical Residues: 198 aa
Alignment Score (without introns) 10187


MG04166.1 	------MKIIIAGAG~MGGLGCALSLARKGFKHIEVYETASNLGFVGAGIQMAPNMVRIL
NCU08315.1	MTDFRDLRIAIIGAG1MGGLGTALAFAKKGFKNINVYETASNLGFVGAGIQMPPNVVRIL
FG05063.1 	MADIKDLHIAIMGAG~MGGLGAALALAKRGFKHIDVYETASNLGFVGAGIQMPPNVGRIL
AN4576.1  	-MAPQDLRVAILGAG1MGGLTSALALAEKGFKHIDIYETASDLGFVGAGIQLAPNMARVL
          	     .::: * *** ****  **::*.:***:*::*****:*********:.**: *:*

MG04166.1 	DRLGCWRGSGIEKEATNVKETSIRQ1GSTNEELAHVNMPDIAAKYGYPHCTGHRSSLAGG
NCU08315.1	DHLGCWE--EIEKEATDVKETSIRQ1GSTGEELAHVSMPDMREKYGFPHCTGHRSSLASG
FG05063.1 	DRLGCWN--DIEKEATRVAGSSIRQ1GSTNEELAHVDMPDLKGQYGYSHLCGHRSSLAGH
AN4576.1  	DKLGVWK--AIEAEAVNIEETSVRV1GASNSELAHVDLHYIEPTYGYKHMVGHRYSLANG
          	*:** *.   ** **. :  :*:*  *::..*****.:  :   **: *  *** ***. 

MG04166.1 	MYEACKKE-PAITFHFGTSVVGVSNWGPA---KPHVTVRSAGADEETKDVEADVVLAADG
NCU08315.1	LYNGCLKE-PAIKFNFVTSLVKVLSWGP----KPRILLKSRAQD-EPFEVEADILLASDG
FG05063.1 	MYEACKKQ-DSIKFHFSTSLVEVETFS------PKVSFKLKPRDGEPYTSQADILLGADG
AN4576.1  	LYQGCLKHSDKITFHFGTSVTEVHSFGRGTQPKPSFTVTPRDSSKQPYTVHADILLGADG
          	:*:.* *.:  *.*:* **:. * .:. .:...* . .     . :.   .**::*.:**

MG04166.1 	IKSVVRREMLARYGAE--PEEEDTGQACYRIMLTRAEMEAI--GEPELLELMDSDLVVRW
NCU08315.1	IKSVTRTQMLSELGLM--SEEEDTGQAAYRIMLHRDQMKG----DPEMLALLDCDTVVRW
FG05063.1 	IKSVTRSQILQQVDAT--PEEAETGQAAYRIMLNREDMAH----DPELLALIDSDEVVRW
AN4576.1  	IKSVTRVAMLNDLAISPVPAVKDTNQAAYRIMIHKDQIESDPNADPELKALLASNKVVRW
          	****.*  :*      . .   :*.**.****: : ::   ...:**:  *: .: ****

MG04166.1 	IGEKRHIIAYSVAGKSIYNLSTTQPDVNFASGPTSTYTTRASKEAMLKVFEDFCPLVQKM
NCU08315.1	IGERRHIIAYPVSGHTIYNLSTAHPDTNFAGATNATYTTRGEKKAMFEVFGTFCPLVHRM
FG05063.1 	VGEKRHIIAYSIADKSIYNLSTAHPDENFAGAPSITYTTKGSKEVMLKVFDTFCPLVQKM
AN4576.1  	IGEKRHIIAYPVSNNTIYNLSTTQPDTNFAAATNATYTTKGSKSAMLGVFADFCPLVQRM
          	:**:******.::.::******::** ***.... ****:..*..*: **  *****::*

MG04166.1 	LNMVPSGDVCEWKLRQHKTLPTWCDGPVALLGDACHPTLPHLSQGAAMAIEDGAVIAEVL
NCU08315.1	LDLVPEGEVCEWKLRIHKTLPTWVHGSVALLGDACHATLPHLSQGAAMAIEDGAVLAECV
FG05063.1 	LNLVPEGEVCEWRLRMYKPLPTWTHGAVALLGDACHPTLPHLSQGAAMAIEDGSTIAEVL
AN4576.1  	LNYVPEGEVCEWKLRTHEPLPTWVDGCTALVGDACHPTLPHLAQGAAQAIEDGAVLAVVL
          	*: **.*:****:** ::.**** .* .**:*****.*****:**** *****:.:*  :

MG04166.1 	SRLPPG-EVDPVQVGRCLKVYEQLRKDRTSELVSLAAMSARALHLGDGKAKEERDRQFAE
NCU08315.1	SRIPADKLDDAETVTKTLKVFEMLRKPYTSTLVDLASHSGRVLHLGEGKAKEERDRLFRD
FG05063.1 	CLAP---DTKPETIAKCLKVYEQSRKEWTSSLVEMAYLSGRTLHLGEGKAKEERDRMFKE
AN4576.1  	SKLP---STDTESINKALKVYEMVRKDRAYALVELAAASGRALHLGEGAAKEERDKQFAA
          	.  *     ..  : : ***:*  **  :  **.:*  *.*.****:* ******: *  

MG04166.1 	ARAKGGAPVPDKWASPEVQKMIYSHDCVQHAAENYDGILANMS-----
NCU08315.1	NGKSG--VVPDKWASPDVQKMIYSYDCVKEVIGKFDEVFASIA-----
FG05063.1 	HKLSG--SVPDKWTSPDVQKKIYSNDCVAKVRDEFETLFAAV------
AN4576.1  	LKAGKG-PVPDKWADADVQREIYGFDCIKEAEQMFEEYFGAAAATATA
          	     .  *****:..:**: **. **: ..   ::  :.  ::::::
```
